# *Drosophila Argonaute-1* is critical for transcriptional cosuppression and heterochromatin formation

**DOI:** 10.1007/s10577-012-9279-y

**Published:** 2012-04-03

**Authors:** Sreerangam N. C. V. L. Pushpavalli, Indira Bag, Manika Pal-Bhadra, Utpal Bhadra

**Affiliations:** 1 Functional Genomics and Gene Silencing Group, Centre for Cellular and Molecular Biology, Uppal Road, Hyderabad, 500007 India; 2 Centre for Chemical Biology, Indian Institute of Chemical Technology, Hyderabad, 500007 India

**Keywords:** Argonaute-1, Heterochromatin, Polycomb, Transcriptional gene silencing, *Drosophila*

## Abstract

Argonaute-1 (*Ago-1*) plays a crucial role in gene regulation and genome stability via biogenesis of small non-coding RNAs. Two “Argonaute” family genes, *piwi* and *Ago-2* in *Drosophila* are involved in multiple silencing mechanisms in the nucleus, transgene cosuppression, long-distant chromosome interaction, nuclear organization and heterochromatin formation. To investigate whether *Ago-1* also plays a similar role, we have generated a series of *Ago-1* mutations by excising *P* element, inserted in the *Ago-1* promoter (*Ago-1* ^*k08121*^). AGO-1 protein is distributed uniformly in the nucleus and cytosol in early embryos but accumulated predominantly in the cytoplasm during the gastrulation stage. Repeat induced silencing produced by the mini-white (*mw*) array and transcriptional cosuppression of non-homologous transgenes *Adh-w/w-Adh* was disrupted by *Ago-1* mutation. These effects of *Ago-1* are distict from its role in microRNA processing because Dicer-1, a critical enzyme for miRNA biogenesis, has no role on the above silencing. Reduction of AGO-1 protein dislodged the POLYCOMB, EZ (enhancer of zeste) and H3me3K27 binding at the cosuppressed *Adh-w* transgene insertion sites suggesting its role in Polycomb dependent cosuppression. An overall reduction of methylated histone H3me2K9 and H3me3K27 from the polytene nuclei precisely from the *mw* promoters was also found that leads to concomitant changes in the chromatin structure. These results suggest a prominent role of *Ago-1* in chromatin organization and transgene silencing and demonstrate a critical link between transcriptional transgene cosuppression, heterochromatin formation and chromatin organization. We propose *Drosophila Ago-1* as a multifunctional RNAi component that interconnects at least two unrelated events, chromatin organization in the nucleus and microRNA processing in the cytoplasm, which may be extended to the other systems.

**Electronic supplementary material:**

The online version of this article (doi:10.1007/s10577-012-9279-y) contains supplementary material, which is available to authorized users.

## Introduction

RNA interference (RNAi) and silencing mechanisms control gene expression at both transcriptional and post-transcriptional level (Hanon 2002; Mazke and Birchler 2005). Small regulatory RNA molecules, siRNA and endogenous miRNA, play a central role in RNAi as a guide. During post-transcriptional gene silencing (PTGS) different classes of small regulatory RNA, are loaded onto the RNA induced silencing complex (RISC) containing a conserved Argonaute protein, which binds siRNAs and also directly cleaves target mRNA sequences (Ghildiyal and Zamore 2009). On the other hand, RNAi can also trigger DNA methylation and/or chromatin modifications that lead to transcriptional silencing and heterochromatin formation. These RNA-directed chromatin modifications are best studied in fission yeast *Schizosaccharomyces pombe* (Bernstein and Allis 2005; Buhler and Moazed 2007).

In fission yeast, siRNAs are derived from centromeric repetitive DNA (*CenH)* and the formation of heterochromatin at these repeats requires components of the RNAi pathways that initiate H3K9 methylation followed by the recruitment of SWI6*,* a *Drosophila* homologue of HP1 (Hall et al. 2002). The Ago-1 containing RNA-induced transcriptional silencing complex is required for establishing heterochromatin assembly at the centromeres (Verdel et al. 2004, Hall et al. 2002). These results suggest that *Ago-1* is involved in the establishment of chromatin organization especially via histone tail modifications. It is also plausible that similar function of *Ago-1* is conserved in higher eukaryotes*,* along with its contribution in miRNA biogenesis.

It has been shown that few RNAi factors are involved in repeat induced gene silencing and heterochromatin formation at the centromere and telomere repeats in *Drosophila*. The modifiers of centromeric silencing often do not influence telomeric silencing (Boivin et al. 2003), because centromeric heterochromatin is functionally distinct from the condensed track of chromatin located elsewhere. *piwi,* an *Ago* family gene suppresses centromeric and pericentric silencing but caters an opposite effect in telomeric silencing associated with PIWI bound small RNAs (piRNA) (Yin and Lin 2007). Further, a physical interaction between PIWI and HP1 proposed an alternate pathway for heterochromatin silencing (Brower-Toland et al. 2007) that is different from conventional H3me2K9 dependent heterochromatin assembly (Fanti et al. 1998). The PIWI protein is associated with PIWI-interacting RNAs (piRNAs) strongly and interacts with heterochromatin protein 1a (HP1a) (Brower-Toland et al. 2007). Later it was found that heterochromatin formation is independent of piRNA or endo-siRNA pathways (Moshkovich and Lei 2010). Apart from heterochromatin silencing, *piwi* and *aubergine* are also involved in certain aspects of Polycomb dependent transgene cosuppression and PTGS in *Drosophila* (Pal-Bhadra et al. 2002; Aravin et al. 2004). Both *piwi* and *Ago-1* were also shown to be required for clustering of PRE containing Pc-G targets in the embryonic nuclei as shown by the overlapping Pc-G and PIWI or Pc-G and AGO-1 binding (Grimaud et al. 2006). It is reasonable to anticipate that similar to *piwi*, *Ago-1* might be involved in *PcG*-mediated transgene cosuppression and PTGS in flies. The present study was carried out to investigate the role of *Ago-1* on transgene silencing. Here, we have generated a series of *Ago-1* mutations. Using these mutations, we have evaluated the exact role of microRNA processor *Ago-1* in chromatin packaging, transcriptional gene silencing and the recruitment of the chromatin bound proteins at the transgene insertion sites.

## Materials and methods

### Fly stocks and generation of *Ago-1* excision lines

Flies were cultured in standard *Drosophila* food media at 25°C. Majority of the fly markers are noted in FLYBASE (http://flybase.org) unless otherwise described. Mutations of the *Argonaute-1* gene, *{y* ^*1*^ *w* ^*67c23*^ *; P{w[+mC] = lacW}Ago-1* ^*k08121*^ */CyO)* were obtained from Bloomington Stock Centre (http://flystocks.bio.indiana.edu, Williams and Rubin 2002). Two independent alleles *Ago-1* ^*72*^ (*Ago-1a*) and *Ago-1* ^*99*^ (*Ago-1b*) were generated earlier by the mobilization of single *P* element on the *Ago-1* regulatory region using a transposase stock *(Δ2-3Sb/TM3 Ser*, Grimaud et al. 2006). Using same genetic schemes, a series of imprecise excision lines were generated that were primarily screened against the loss of adult eye colour produced by the *mini-w* marker gene of the *P* element (Fig. S1).

**Fig. 1:**
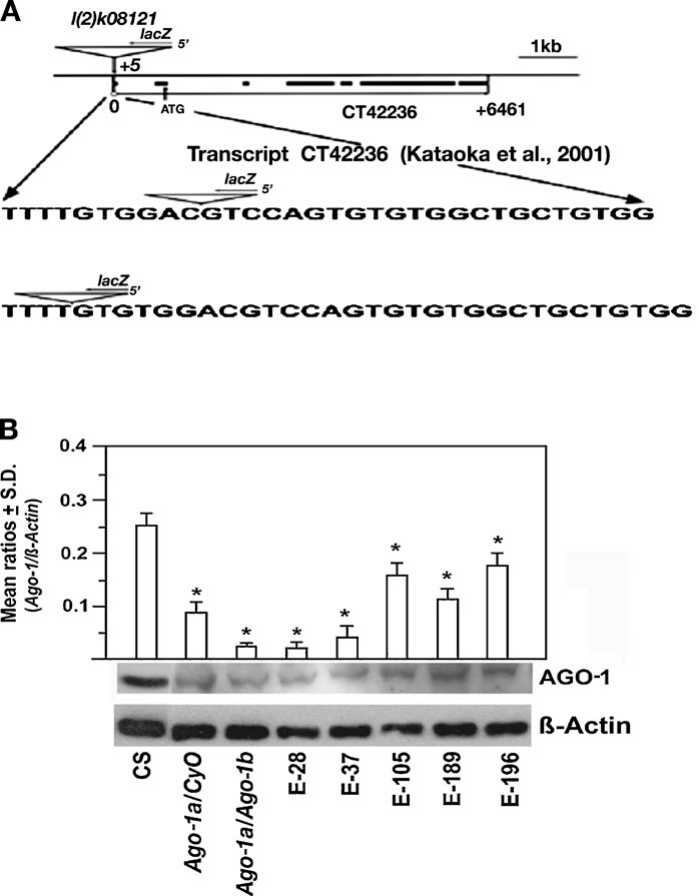
Generation of excision lines of *Ago-1* and their expression by western blot analysis were generated. **a** A schematic diagram showing structure of the 5′ of the *Ago-1* gene with *P* element insert (*inverted triangle*). The sequences of the insertion site of the *P* element (*blank triangle*) as reported earlier (*top*) and the present study (*bottom*) were marked. **b** Western blot analysis of AGO-1 protein (Kataoka et al. 2001) extracted from adult female flies carrying *Ago-1* excision mutation. Wild-type, heterozygous and heteroallelic *Ago-1* mutant combinations were used and β-actin acts as gel loading control. Three independent Western blots were conducted for each genotype. Each blot showed a similar profile, first lane (*CS*) was taken from a different lane of the same gel and merged

Initially, we have generated 67 independent *Ago-1* mutations by excising the *P*-element of two *Ago-1* independent *P* element insertion alleles (*Ago-1* ^*72*^ and *Ago-1* ^*99*^) (Table S1). As described earlier the *P* element in *Ago-1* ^*72*^ (*Ago-1a*) was located to 5 bp upstream to the transcriptional start site (Fig. 1a), whereas *P* element in *Ago-1* ^*99*^ (*Ago-1b*) is inserted into 27 bp away from transcriptional start site of the *Ago-1* promoter.

The *Ago-1* ^*45*^ is derived from *Ago-1* ^*72*^ in which excision of the *P* element has retained one *P* foot and has deleted the neighboring sequence, including the transcriptional start site (Grimaud et al. 2006). We recovered three more excision lines (*e-28*, *e-37*, *e-105*) from the same cross in which *P* element retains both feet but eliminate *mini-w* marker genes. Similarly, three excision lines (*e-189*, *e-196*, *e-242*) derived from *Ago-1* ^*99*^ (*Ago-1b*) insert were selected. They also retain the feet of the *P* element but *mini-w* sequence is deleted. The stocks with these mutations were balanced over *CyO* chromosome. Females of each excision lines, recovered from excision of *P* element from two parental stocks (*Ago-1a* and *Ago-1b*) were further crossed to the heterozygous males from *Ago-1* deficiency stock [*Df (2R)50 C-107/CyO*]*.* We did not rescue any viable homozygous escapers. It indicates that *Ago-1* sequence is functionally disrupted in those alleles (Table S2). Six of these isolated stocks (*e-28*, *e-37*, *e-105*, *e-189*, *e-196*, *e-242*) were allelic to each other as they failed to complement fully the recessive lethality. Only in rare combinations they generate heteroallelic escapers at low frequencies (Table S3), which are used for further studies.

### Genetic crosses

The *w-Adh* transgenic stock contains 2.5 kb *w* regulatory sequence fused to 1.9 kb *Adh* structural gene under *Adh* ^*fn6*^ mutant background. The reciprocal *Adh-w* stock contains a 6.2 kb *w* structural sequence joined to 1.8 kb *Adh* promoter fragment in the *w* minus background (Pal-Bhadra et al. 1997, 2002). Therefore *w-Adh* and *Adh-w* transcripts in the transgenic stocks are the sole source of the *Adh* and *w* mRNA, respectively.

To examine the effect of *Ago-1* mutation on the *Adh-w/w-Adh* transgene silencing (Pal-Bhadra et al. 1999), two independent *Ago-1* excision alleles (*Ago-1* ^*e-28*^ and *Ago-1* ^*e-37*^) were crossed to produce adult heteroallelic escapers with white eyes. One *Ago-1* allele (*Ago-1* ^*e-28*^) was combined with a single *Adh-w* transgene inserted in the X chromosome. Subsequently, another *Ago-1* ^*e-37*^ allele was combined with *w-Adh* transgene inserted in the chromosome 3. The females *Adh-w/Adh-w*; *Ago-1* ^*e-28*^ */CyO* were further crossed to the *Ago-1* ^*e-37*^ */CyO*; *w-Adh/w-Adh* males. The eye colour of the *Adh-w/Y*, *w-Adh/+* male siblings that are heterozygous (*Ago-1* ^*e-28*^ */+* or *Ago-1* ^*e-37*^ */+*) and/or hetero-allelic for the *Ago1* mutations (*Ago1* ^*e-28*^ */Ago-1* ^*e-37*^) were compared. The eye pigment as well as *w* and *Adh* transcripts from each genotype were also estimated (Pal-Bhadra et al. 1999).

To study the effect of the *Ago-1* mutation on the repeat induced silencing, three *mini-w* transgenic stocks were used. The *mw 6-2* is a transgenic *P[lacW]* stock, in which single copy of *Drosophila mw* transgene was inserted at the 50 C2 site of the chromosome 2, which is away from the centric and pericentric heterochromatin (Dorer and Henikoff 1994). *BX2* stock contains seven tandem copies of same *mw* transgene inserted at the same location and *DX1* contains seven tandem copies of *mw* transgenes in which one copy is inverted. The *BX2* and *DX1* stocks show variegated eye colour phenotype. All these stocks were crossed separately with two *Ago-1* excision (*Ago-1* ^*e-28*^, *Ago1* ^*e-37*^) alleles.

To test the effect of *Ago-1* mutation on the position effect variegation, the same *Ago-1* (*Ago-1* ^*e-28*^, *Ago-1* ^*e-37*^) alleles were combined with *y* ^*3p*^ variegated mutation and *In(1)w* ^*m4h*^ inverted chromosomes separately (Bhadra et al. 1997). In *In(1)w* ^*m4h*^ stock, a large inversion juxtaposes *w* gene next to the centromeric heterochromatin.

### Pigment assay

For eye pigment assay, 50 heads of adult male flies were dissected manually from each (3–4 days post-eclosion) genotype and homogenized in 0.5 ml of 0.01 M HCl in ethanol. The homogenate was incubated at 4°C overnight and further warmed at 50°C for 5 min. The samples were centrifuged, and the OD of the supernatant was recorded at 480 nm (Ephrussi and Herold 1944).

### Fly genomic DNA isolation and PCR

Genomic DNA was extracted from each *Ago-1* excision line and parental *P* strains as described earlier (Huang et al. 2000). To determine the sequence deleted from the ends of the *P(lacW)* element, the sequence at the junction of the *P* element and *Ago-1* promoter from each excision line was amplified using a *P* element specific forward primer (forward primer-5′-ACAACCTTTCCTCTCAACAAGC-3′) and *Ago-1* gene specific reverse primer (reverse primer-5′-TTTTTGTGCACCAAACACGTTCG-3′) The polymerase chain reaction (PCR) product was sequenced (Applied Biosystems 3730) using gene-specific reverse primer as noted above. The sequences were aligned with *P(lacW)* and fly genomic sequences carrying wild type *Ago-1* gene using NCBI BLAST program.

### RT-PCR and Western blot analysis

For semi-quantitative reverse transcription Polymerase chain reaction, 1 μg of clean RNA, isolated by the TRIzol method (Invitrogen, USA) was used for each reaction. Ten picomoles of the gene-specific reverse primer and *18S rRNA* reverse primer (internal control) were used for converting RNA into cDNA using superscript reverse transcriptase enzyme. The cDNA was amplified using standard PCR. The PCR products were loaded on 1% agarose gel. The primers used for *Ago-1* and *18S rRNA* are as follows:

For *Ago-1*

- Forward primer (FP): 5′-ATA ATA CCT CGT TCG CAA CT-3′
- Reverse primer (RP): 5′-TAA TGA CAA CAA GGA TGC AA-3′

and *18S rRNA*

- FP: 5′-CCT TAT GGG ACG TGT GCT TT-3′
- RP: 5′-CCT GCT GCC TTC CTT AGA TG-3′

For Western blot, nearly 50 adult flies were homogenized in lysis buffer [6% SDS, 1 mM EDTA, 2 mM PMSF, 10 μg/ml aprotinin, 10 μg/ml leupeptin, 10 μg/ml pepstatin] and boiled at 95°C for 5 min. The amount of total proteins of each genotype was measured using Bradford assay (Bradford 1976). Western blot was carried out using anti-rabbit AGO-1 (1:500) antibody (Kataoka et al. 2001). The blots were re-probed with β-actin antibody (1:2,000) as a gel loading control.

The basic histone proteins from wild-type and *Ago-1* mutant flies were extracted following acid extraction method (Pal-Bhadra et al. 2007). Western analysis was carried out in two separate sets of blots using anti-rabbit H3me2K9 (1:3000) and anti-rabbit H3me3K27 (1:1500) antibodies. The blots were re-probed with anti-mouse histone H3 (1:2000) antibody that serves as gel loading control.

### Northern blot analysis

Total RNA was extracted from adult flies using Trizol. Northern blots were carried out as described earlier (Pal-Bhadra et al. 1997). Fifteen microgras per lane of RNA was used for Northern gel and probed with P^32^ radioactive labelled *w* and *Adh* antisense RNAs. The blots were reprobed with antisense *ß-tub* RNA as a gel loading control.

### Embryo staining

*Drosophila* embryos were collected in small embryo collection baskets and washed thoroughly with water. The embryos were dechorionized with 50% commercial bleach and fixed with 4% paraformaldehyde. Fixed embryos were transferred to glass scintillation vials containing 1.6 ml of 0.1 M Hepes pH 6.9, 2 mM magnesium sulphate, 1 mM EGTA and 0.4 ml of 20% paraformaldehyde. The vials were gently stirred to maintain an effective emulsion of organic and water phases for 15 to 20 min. The lower phase was separated and 10 ml methanol was added. The embryos were therein transferred to a solution of 90% methanol + 10% 0.5 M EGTA followed by washes in PBT (PBS + 0.1% Tween 20) three times of 5 min each. Thereafter, the embryos were treated with RNase A. For blocking, embryos were incubated in PBT containing 2% goat or animal serum for 30 min followed by overnight incubation with primary rabbit anti-AGO-1 (1:50) (Kataoka et al. 2001) and rabbit anti-Pc (1:100 dilution) (Grimaud et al. 2006) antibodies at 4°C. The embryos were further incubated in FITC conjugated or Cy5 conjugated secondary antibodies (1:50 dilution) for 2 h at room temperature followed by washes in PBS for two times of 5 mins each. The embryos were then mounted in propidium iodide containing VECTASHIELD Mounting Media. The photographs of intact embryos and the Z-sections of embryos were viewed under confocal microscope (Zeiss - 20X and 100X lens [LSM510]).

### In situ hybridization

In situ hybridization (FISH) was carried out as described earlier (Pal-Bhadra et al. 1999) to determine the cytological location of *Adh-w* at 16B and *mini-w* at the 50 C2 region.

### Polytene chromosome preparation

Polytene chromosomes were prepared from the salivary glands of the well-fed third instar *Ago-1* mutant and wild-type larvae (*Canton S*) as described earlier (Pal-Bhadra et al. 2002). The chromosomes were incubated with anti-H3me2K9 (1:25) (Upstate, USA), anti-H3me3K27 (1:25) (Upstate, USA), anti-EZ (1:100) (Grimaud et al. 2006) and anti-Pc (1:50) (Grimaud et al. 2006) antibodies, respectively.

### Chromatin immunoprecipitation

Chromatin immunoprecipitation (ChIP) assay was carried out as per protocol described earlier (Cavalli et al. 1999) using H3me2K9 and H3me3K27 antibodies (Upstate, USA) from the immunoprecipitated chromatin of the third instar wild-type and *Ago-1* mutant larvae. The primer sets used for amplification of specified regions of the *w* gene are summarized in Table S4. Relative enrichments were calculated as the ratio of product (*w* promoter/*w* second exon) in IP over input. Histograms represent data from three biological replicates analysed in parallel.

## Results

### Expression of *Ago-1* excision alleles

To test whether *Ago-1* excision alleles have reduced transcripts and/or protein, semi-quantitative RT-PCR and Western blot analysis was carried out using six excision lines (*e-28*, *e-37*, *e-105*, *e-189*, *e-196*, *e-242*). Total RNA was isolated from each line and quantitative RT-PCR was carried out. Amplified ribosomal (*18S rRNA*) RNA from the same sample served as an internal control. The relative ratio of *Ago-1/18S rRNA* from each line showed a dramatic reduction relative to the same ratio (*Ago-1/18S rRNA*) from the parental *w* minus flies (Fig. S2). The difference of *Ago-1* mRNA level in each line revealed that excision lines show marked reduction in *Ago-1* transcript.

Since excision lines are recessive lethal, only adult flies heterozygous for each *Ago-1* line and a heteroallelic combination of two parental mutations (*Ago-1a*, *Ago-1b*) were further used. Total protein from adult flies of each allele was isolated and Western blot analysis was carried out using AGO-1 antibody (Kataoka et al. 2001), while β-actin served as a gel loading control. Similar to mRNA level, each excision line showed a profound reduction in the AGO-1 protein relative to wild type (Fig. 1b) as described earlier (Okamura et al. 2004).

### Distribution of AGO-1 protein in *Drosophila* embryos

**Fig. 2:**
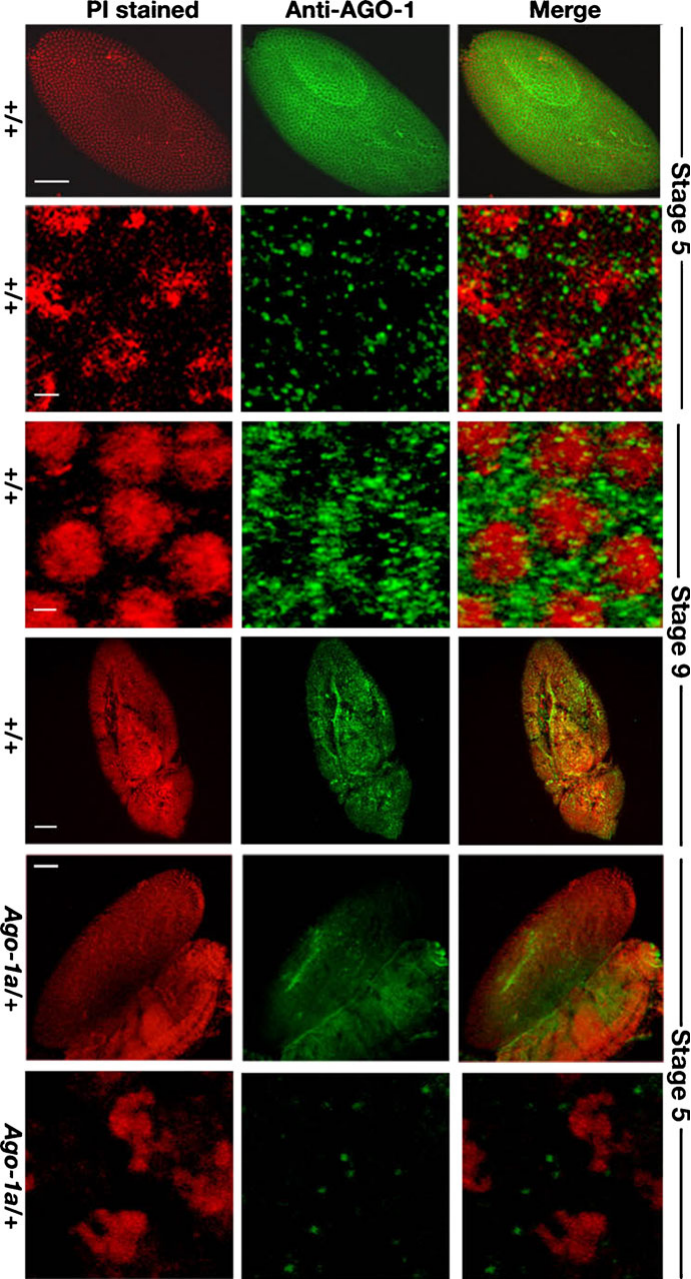
Distribution of AGO-1 protein in the wild-type *Drosophila* embryos. The AGO-1 protein is uniformly distributed during the blastoderm stage (stage 5) significant above the background level (enlarged view in few nuclei), while its accumulation is intense in the cytoplasm during the gastrula stage (stage 9 embryos and enlarged view). Loss of AGO-1 protein in heterozygous *Ago-1* combinations (*Ago-1a*/+) has shown a dramatic loss of AGO-1 protein localization either in embryos or in an enlarge view of the nuclei (stage 5). Scale 50 μm in embryos and scale 10 μm in enlarge nuclei

To show AGO-1 protein distribution during development, we immunostained early syncytial blastoderm and mature gastrula embryos with anti-AGO-1 antibody (Kataoka et al. 2001). A moderate amount of AGO-1 protein was accumulated initially in the nucleus and cytoplasm of the blastoderm embryos, nearly 2.5 h after egg laying (AEL) which is above the background level (Fig. 2; stage 5). However, in *Ago-1* heterozygous mutant embryos localization of the AGO-1 protein was reduced (Fig. 2; stage 5). We further compared differential distribution patterns of AGO-1 protein in the mature gastrula stages. A marginal increase of staining intensity was noticed at the anterior end of the ventral furrow, the midgut plate and posterior part of the elongated germ band during the gastrulation stage nearly 3–4 h AEL (Fig. 2; stage 9). In gastrula embryos, the protein was mostly localized in the cytoplasm of the cells (Fig. 2; enlarged view, stage 9), which is distinct from the uniform distribution of AGO-1 protein in the nucleus and cytoplasm during the blastoderm stage (stage 5, enlarged view). This clear shift of AGO-1 protein from nucleus to cytoplasm is unique for RNAi factor because majority of the RNAi proteins are localized in the cytoplasm.

### Role of *Ago-1* mutation on transcriptional transgene silencing

**Fig. 3:**
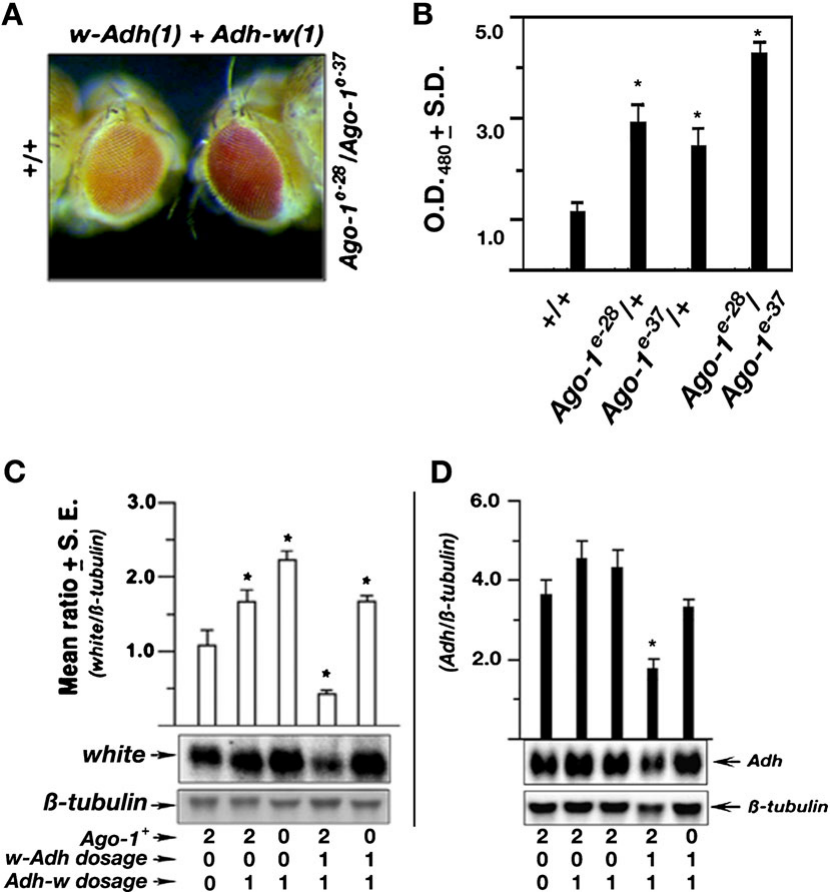
Role of *Ago-1* in transgene induced silencing. **a** The eye colour of *Adh-w*/Y transgenic flies carrying one copy of reciprocal *w-Adh* constructs that are wild type (+/+) and heteroallelic (*Ago-1* ^*e-28*^ */Ago-1* ^*e-37*^) for *Ago-1* mutation were shown. All flies have *w* minus background. The transgene copy number was noted in the parenthesis. **b** The adult eye pigment level of the *Adh-w/Y*; *w-Adh/+* flies having zero, one and two copies of *Ago-1* mutation were estimated from three independent sets of experiments. The relative ratios were presented in a bar diagram. The values marked with *asterisks* are significantly different from +/+ control values (*P* < 0.05). **c**, **d** Autoradiogram showing northern blot hybridization probed with *w* and *β-tub* antisense RNA (*left*) and rehybridized with *Adh* and *β-tub* antisense RNAs. The graph depicts the mean values of results obtained from three independent blots. The copy number of each construct or *Ago-1* ^*+*^ alleles is noted at the bottom. All flies have *w* minus and *Adh* ^*fn6*^ background. The values marked with asterisks are significantly different from the control values (*P* < 0.05)

Earlier, we have shown that single copy of *w-Adh* is sufficient to reduce the expression of one copy of *Adh-w* transgene close to 50% of its normal level (Pal-Bhadra et al. 1999). To examine the role of *Ago-1* mutation on the *Adh-w/w-Adh* transgene cosuppression, the expression of *Adh-w* transgene was carried out in the *w-Adh/Adh-w* flies carrying *Ago-1* heterozygous or heteroallelic mutations (*Ago-1* ^*e-28*^ */Ago-1* ^*e-37*^). The *Ago-1* excision alleles produced a considerable number of heteroallelic *w* minus progeny. Therefore, *Adh-w* transgene is the sole source for red-eye pigment and *w* transcripts when heteroallelic *Ago-1* escapers are combined with *Adh-w* transgene. The effect of *Ago-1* mutation on *Adh-w* transgenes was detected in the adult eye colour phenotype and by quantitative northern blot analysis using *w* and *Adh* radiolabelled probes. Initially, two independent stocks *Adh-w/Y*; *Ago-1* ^*e-28*^ */CyO*; *w-Adh/+* and *Adh-w/Y*; *Ago-1* ^*e-37*^ */In(2LR)Gla*; *w-Adh/+* were generated by employing multiple crossing schemes (Supplementary Materials). Each *Ago-1* allele or alleles in heteroallelic combination did not show any change in the eye colour of the *Adh-w* males in the absence of the *w-Adh* construct*.* However, a mild but consistent increase in the eye colour of *Adh-w/w-Adh* flies in the heterozygous *Ago-1* mutant flies was observed relative to the wild type that was restored close to a normal level in heteroallelic *Ago-1* mutant (*Ago-1* ^*e-28*^ */Ago-1* ^*e-37*^) flies (Fig. 3a, b). Thus, an elevation in the *Adh-w/w-Adh* eye colour in the presence of *Ago-1* mutation indicates that transgene silencing is relieved from the adult eyes, which suggests that *Ago-1* mutation represses non-homologous transgene cosuppression (Pal-Bhadra et al. 1999).

Further, the *w* transcript levels of the same genotypes were also measured by Northern blot hybridization. A combination of *Adh-w/w-Adh* transgenes produced 50% of the normal *Adh-w* mRNA level (Pal-Bhadra et al. 1999, 2002). The reduced *w* mRNA from the same transgene combination (*Adh-w/w-Adh*) was also restored close to the normal expression level in the presence of same heteroallelic *Ago-1* mutations (Fig. 3c). As expected *Ago-1* mutation shows no effect on the *Adh-w* mRNA alone. Data revealed that similar to eye colour, *Ago-1* mutations reduce *Adh-w/w-Adh* silencing considerably at the transcriptional level.

We further reprobed the same RNA blots with radiolabelled *Adh* antisense RNA to determine the effect on the *w-Adh/Adh* silencing (Fig. 3d). As shown earlier, the amount of *Adh* transcripts was proportionately reduced to the *w-Adh* transgene dosage when one or two copies of *w-Adh* (*Adh-w/Y*; *w-Adh/+* or *Adh-w/Y*; *w-Adh/w-Adh*) were added to the same genotype (Fig. 3d; Pal-Bhadra et al. 1997). However, a marginal increase of *Adh* mRNA level was found in *Adh*-w/Y files in the presence of heteroallelic *Ago-1* combinations. It suggests that difference in genetic background in the parental chromosomes 2 carrying each *Ago-1* allele might provide these changes in *Adh* transcripts.

### Role of *Ago1* on tandem *mini-w* repeats and position effect variegation

**Fig. 4:**
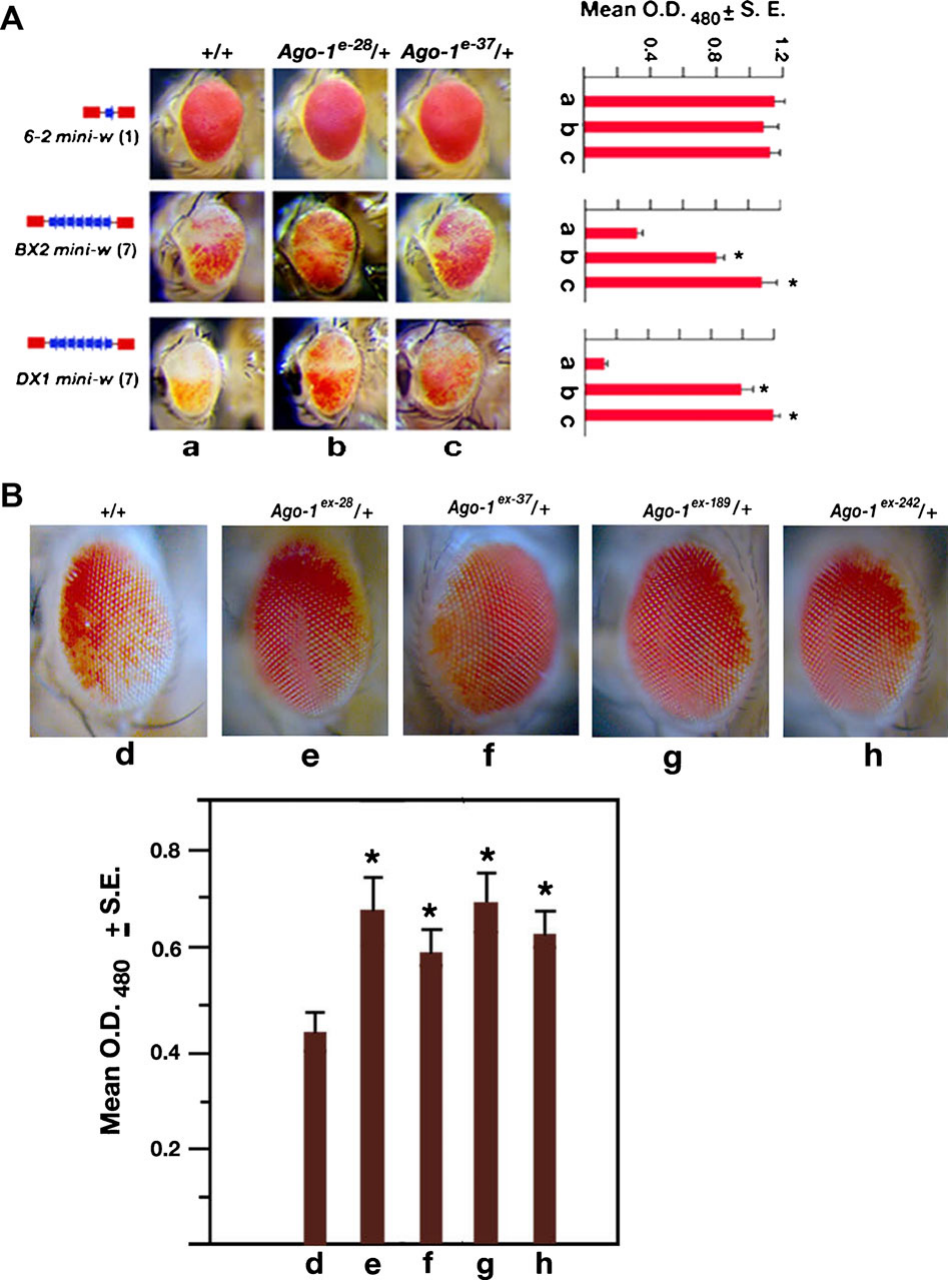
*Ago-1* is required for repeat sensitive silencing and position effect variegation (PEV). **a** The eye colour of the *Ago-1* heterozygous males that carry one copy (*mini-w 6-2*), seven tandem copies (*BX2 mini-w*) or seven copies with one inverted (*DX1 mini-w*) white genes were compared. The number of the *mini-w* copies in the transgenic construct was noted in the parentheses. Mean values (*bar*) of the eye pigment level from triplicate assays of each genotype were reported by a bar diagram with a standard error. **b** *Ago-1* is a weak suppressor of position effect variegation. The eye colour of the adult males carrying one copy of large X chromosomal inversion [*In(1)w* ^*m4h*^] was tested with wild-type or *Ago-1* heterozygous mutations. The level of average eye pigment in the adult eyes, displayed by a histogram was marginally high (28–30%) relative to *In(1)w* ^*m4h*^ flies

A distinct accumulation of HP1 protein and H3me2K9 methylation at the tandem *mini-w* array (*BX2* and *DX1*) and their subsequent disruption by the HP1 mutation suggests that *mini-w* repeats transform into a heterochromatin like structure (Dorer and Henikoff 1994). To analyse whether *Ago-1* has any role on heterochromatin formation, variegated expression of the white eye colour in the *mini-w* repeats (*BX2* and *DX1*) was examined in two heterozygous *Ago-1/+* (*Ago-1* ^*e-28*^ */+* and *Ago-1* ^*e-37*^ */+*) backgrounds. However, we failed to detect the level of variegation in heteroallelic combination because *mini-w* arrays and *Ago-1* mutations located on the chromosome 2. A two- to three folds increase in the eye pigment was observed in different heterozygous *Ago-1* mutant combinations (Fig. 4a) compared to wild type. The amount of eye pigment produced by single *mini-w* insert in the same location remained unchanged by the same *Ago-1* mutant combinations (Fig. 4a). These results suggest that *Ago-1* plays a definitive role in repeat induced *mini-w* silencing.

Similarly, to analyse the role of *Ago-1* on the variegated *Drosophila w* gene expression or PEV, we combined one copy of *Ago-1* mutation with *In(1)w* ^*m4h*^ chromosome, in which the *w* gene is juxtaposed close to the heterochromatin. The *Ago-1* mutation increases the red pigment in the variegated white eye (*w* ^*m4h*^) considerably (28–30% only) compared to the siblings carrying wild-type chromosomes (+/+) (Fig. 4b). This demonstrated that unlike repeat associated silencing, *Ago-1* does not show any dramatic change in the pericentric heterochromatic silencing. Thus, the effect of *Ago-1* mutations on the two different types of silencing indicates that *Ago-1*-dependent silencing machinery is involved primarily in heterochromatin like structure formation.

The effect of *Ago-1* mutations was further analysed on the variegated allele of the *y* (*y* ^*3p*^) gene*.* The *y* gene (*y* ^*3p*^) displays PEV when placed next to heterochromatin by an inversion *In(1)y* ^*3p*^. In addition to *w* ^*m4h*^ suppression, *Ago-1* also reduced the frequency of variegation of the yellow bristles along the anterior margin of the wing blades. The number of wild type and yellow bristles of ten wing blades was counted from *Ago-1* mutant and wild-type flies generated from the same cross. The *Ago-1* mutations suppress yellow bristle variegation (22–27%) above the comparable control level (16%) (Table 1). Though the number of yellow bristle varies from wing to wing, but a consistent increase in the variegation of 6–11% greater in the *Ago-1* flies over the control was found. A consistent but reasonable inhibition of *y* and *w* variegation indicates that *Ago–1* is a weak PEV modifier. Therefore, *Ago-1* affects heterochromatin formation more intensely at the repeat elements rather than at the centromere.

**Table 1:** The effect of *Ago-1* on *y* ^*3p*^ variegation

		No. of triple row bristles^a^		
Genotypes	No. of samples	Yellow^+^	Yellow	Percentage of yellow^+^ bristles
*y* ^*3p*^ */Y; Ago-1a/Ago-1b*	10	21.37 ± 4.14	57.91 + 5.11	27%
*y* ^*3p*^ */Y; CyO/Ago-1b*	10	18.01 ± 2.67	61.87 + 5.91	22%
*y* ^*3p*^ */Y; Ago-1a/CyO*	10	19.34 ± 3.23	59.86 + 3.77	24%
*y* ^*3p*^ */Y; +/CyO*	10	13.67 ± 3.95	64.96 ± 6.14	16%

The degree of *Ago-1* suppression on *y* ^*3p*^ variegation was measured by determining the colour of 80 marginal triple bristles between the first and second cross vein termini of wings. The percentage of suppression is determined by the increased number of yellow^+^ (black) bristles in *Ago-1a/Ago1b* relative to *CyO/+*

^a^Values are mean ± S.D

### *Drosophila* Dicer-1 has no role on *w* mRNA

*Ago-1* is a critical factor for microRNA biogenesis (Diederichs and Haber 2007). Thus, repression of *w* gene silencing in *mini-w* repeats and *Adh-w* transgene in reciprocal constructs (*Adh-w/w-Adh*) by the *Ago-1* mutations may either be operated through miRNA-mediated control or by a miRNA independent pathway. To investigate whether *w* is an immediate target for any *Drosophila* microRNAs which are processed by Dicer-1 and AGO-1 proteins, we tried to predict the targeted microRNAs that bind to the 3′ UTR of the *w* gene using three commonly used *miRGen*, *Targetscan* and *Pictar* predicted tools. Unfortunately, all three predicted tools fail to pick any common miRNA coupled at the 3′ end of *Drosophila w* gene. It predicts that *w* is not a direct target for known *Drosophila* Dicer-1 processed microRNA.

**Fig. 5:**
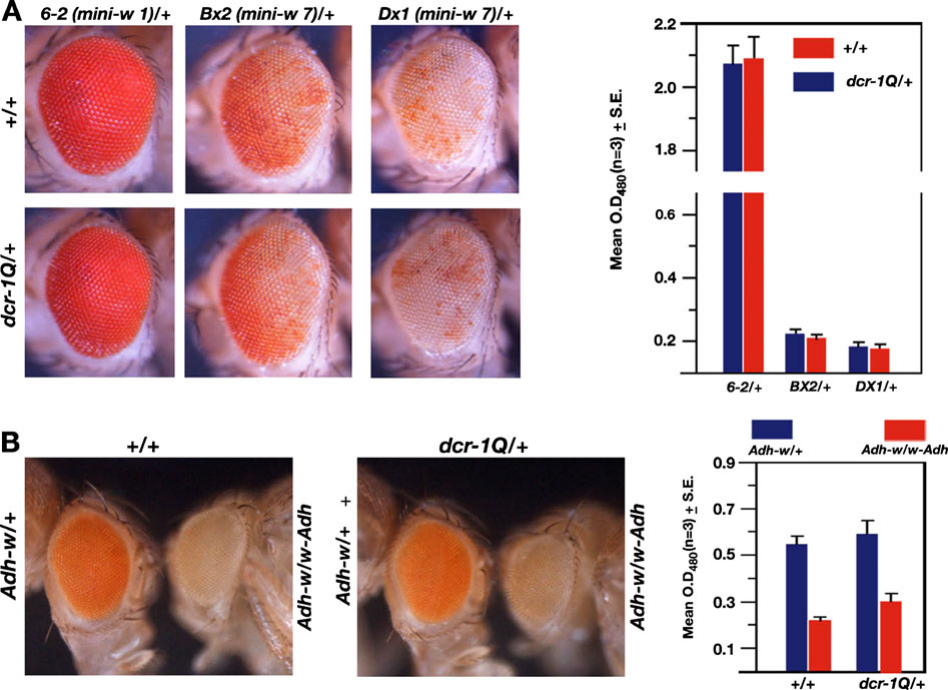
The effect of *dcr-1* mutation on *w* expression. **a** The eye colour of the *dcr-1Q* ^*1147X*^ */+* heterozygous males that carry one copy (*mini-w 6-2*), seven tandem copies (*BX2 mini-w*) or seven copies with one inverted (*DX1 mini-w*) *w* genes were compared. Mean values (*bar*) of the eye pigment level from triplicate assays of each genotype were reported by a bar diagram with a standard error. **b** The eye colour of the adult males carrying one copy of large X chromosomal inversion [*In(1)w* ^*m4h*^] was tested with wild-type or *dcr-1Q* ^*1147X*^ */+* heterozygous mutations. The level of average eye pigment in the adult eyes was presented by a histogram

Further, RNAi effector enzymes Dicer-1 (*dcr-1*) is required for cleavage of pre-microRNA to mature microRNA. Therefore *dcr-1* mutation fails to process functional microRNAs. If *w* gene was controlled by the *dcr-1* processed microRNA, an upregulation of *w* mRNA is expected in the absence of these microRNAs. To test, a loss-of-function *dcr-1* mutation (*dcr-1Q* ^*1147X*^) was used (Lee et al. 2004; Grimaud et al. 2006). The *mini-w* repeats and reciprocal transgenes (*Adh-w/w-Adh*) were combined with one copy of *dcr-1Q* ^*1147X*^ */+* mutation. The presence of *dcr-1Q* ^*1147X*^ mutation does not alter eye pigment level of the flies carrying *mini-w* array or silenced transgene combination (Fig. 5). The amount of eye pigment was assayed in each genotype. An equal expression of eye pigment level in each genotype suggests that *dcr-1* has no role on either *w* gene or *Adh-w* transgene expression. It shows that the mutational effect of miRNA processor (*dcr-1Q* ^*1147X*^ */+*) has no influence on *w* or *Adh-w* RNA. Therefore, *w* gene expression might not be regulated directly through Dicer-1-dependent microRNA pathways, though we cannot rule out the possibility of any indirect microRNA influence on the *w* gene expression.

### *Ago-1* reduces accumulation of H3me2K9 and H3me3K27 methylation

**Fig. 6:**
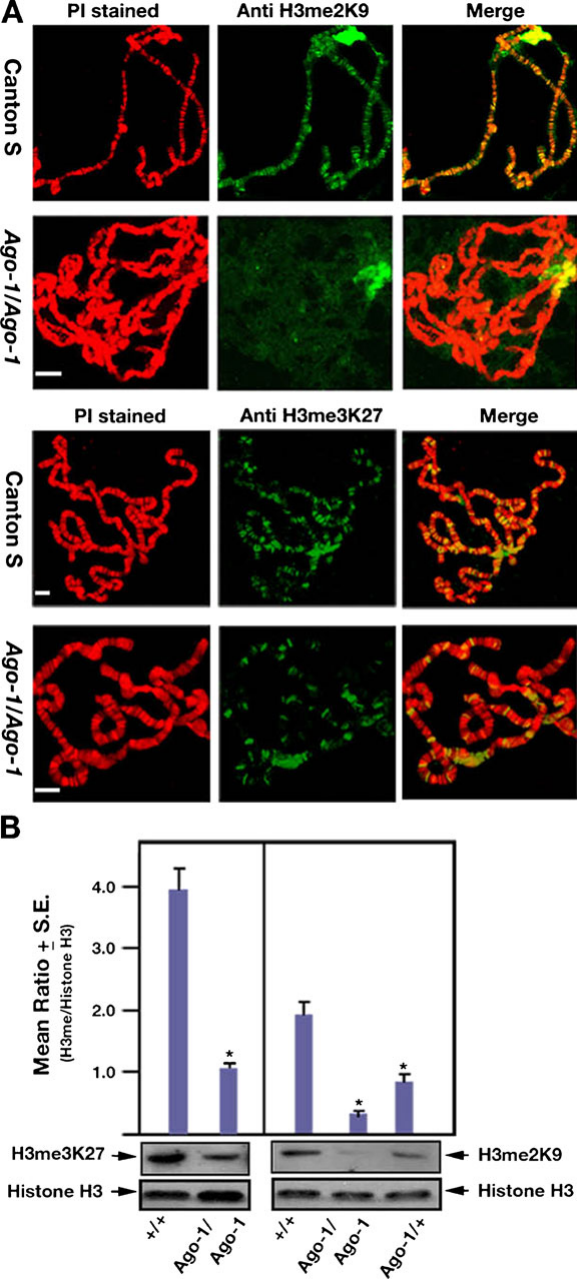
Loss of H3me2K9 and H3me3K27 chromosomal binding by the *Ago-1* mutations. **a** Polytene chromosomes were stained with the same above-mentioned antibodies in *Canton S* and *Ago-1* heteroallelic mutant (*Ago-1a/Ago-1b*)*.* The effect of *Ago-1* on H3me2K9 binding on the polytene chromosomes was more intense than the H3me3K27. The H3me2K9 binding was almost eliminated from the euchromatic region but has minimal effect on the intense accumulation at the chromocentre. Scale, 5 μm. **b** Total histone protein was isolated from the control (*Canton S*) and heteroallelic *Ago-1* mutant *(Ago-1a/Ago-1b)* flies and Western blot analysis was carried out using H3me2K9 and H3me3K27 antibodies. A substantial decrease in the level of the H3me2K9 and H3me3K27 in the presence of *Ago-1* heteroallelic mutations was presented by a histogram depicted from three separate assay

To analyse the contribution of *Ago-1* on heterochromatin we have immunostained polytene chromosomes of third instar larvae carrying wild type and heteroallelic *Ago-1* mutant alleles using H3me2K9 and H3me3K27 antibodies. In wild-type larvae, an intense accumulation of H3me2K9 and H3me3K27 antibodies was found in the chromocenter, telomere and nearly (40–50) methylation sensitive sites at the chromosomal arms as shown earlier (Pal-Bhadra et al. 2004; Fig. 6a). The binding was consistently disrupted from the majority of the chromosomal sites by the loss of AGO-1 protein. Overall, the reduction of H3me2K9 from the euchromatin sites by the *Ago-1* mutation is more pronounced than the loss of H3me3K27 binding (Fig. 6a). At the same time, we also estimated the level of H3me2K9 and H3me3K27 methylation by Western blot analysis. A significant reduction in the H3me2K9 and H3me3K27 levels was found in the *Ago-1* heteroallelic mutations (Fig. 6b). These results suggest that the expression and chromosomal distribution of H3me2K9 and H3me3K27 methylation is *Ago-1* dependent, thereby indicating that *Ago-1* modulates chromosomal organization by largely modifying histone H3 methylation at lysine residues.

### *Ago-1* delocalizes histone methylation at the *mini-w* promoters

**Fig. 7:**
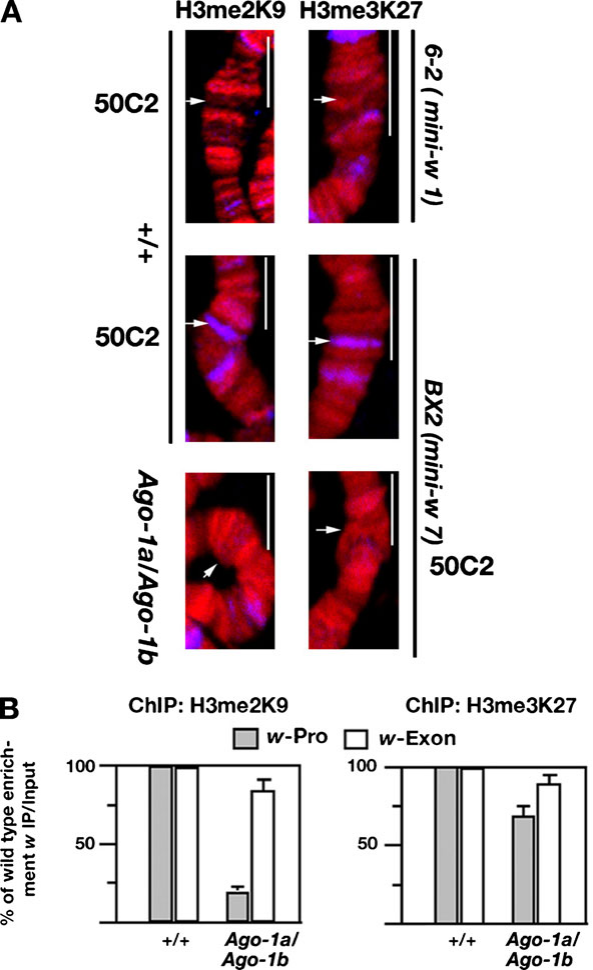
The *Ago-1* mutation diminishes H3me2K9 and H3me3K27 binding in the *mini w* transgenes. **a** Merged chromosomal segments showing localization of the H3me2K9 and H3me3K27 antibodies at the 50 C2 site. A strong accumulation was noticed in the seven copy *mini-w* array (BX2) insertion site in a wild-type background. The binding is abolished by the loss of two copies of *Ago-1* alleles at the same site. The grey colour images are pseudo-coloured. *Arrows* indicate the exact location of *mini-w* insertion sites as detected by the in situ hybridization. Scale 10 μm. **b** Chromatin immunoprecipitation was carried out to compare the accumulation of H3me2K9 and H3me3K27 on the *w* promoter and *w* second exon of the wild-type and *Ago-1a /Ago-1b* larvae. The enrichment of proteins from each amplicon of the *w* locus was measured relative to the *w* second exon and normalized to wild type. The relative ratios from three independent experiments were depicted as bar diagram. The sequences and location of each primer set of the *w* gene were summarized in Table S1 (Supplementary Materials)

We further analysed the binding of histone antibodies on the *mini-w* insertion sites of *BX2* transgenic larvae in a wild-type and/or heteroallelic *Ago-1* mutant background. Two histone methylated (H3me2K9 and H3me3K27) antibodies failed to bind at the insertion site of single copy *mini-w 6-2* transgene, while a consistent binding of the same antibodies was found at the *mini-w* arrays of *BX2* larvae. However, there was no accumulation of H3me2K9 and H3me3K27 at the same *mini-w* sites in the *Ago-1* heteroallelic larvae indicating a loss of histone methylation at the insertion site of the *mini-w* array (Fig. 7a). Thus, the Ago-1 is required for proper targeting of histone H3 tail methylation at the Lysine 9 and Lysine 27 residues at the *mini-w* repeat induced gene silencing.

Next, we performed X Chip analysis using soluble immunoprecipitated chromatins from wild-type and heteroallelic *Ago-1* mutant larvae. The immunoprecipitated DNA was amplified from the promoter or second exon of the *w* gene. The X-Chip assay from the isolated chromatin of the BX2 larvae showed an intense accumulation of H3me2K9 methylation on the promoter site relative to the second exon in wild-type nuclei, whereas accumulation of H3me3K27 at the same promoter site was marginally reduced. However, loss of *Ago-1* showed a sharp reduction in the level of H3me2K9 (Fig. 7b) with a nominal reduction in H3me3K27 protein. A similar degree of reduction was also found in relative quantitative RT-PCR assay. These results suggest that *Ago-1* is critical for deactivation of *mini-w* silencing (Fig. 7b).

### AGO-1 colocalizes with Pc-G proteins

**Fig. 8:**
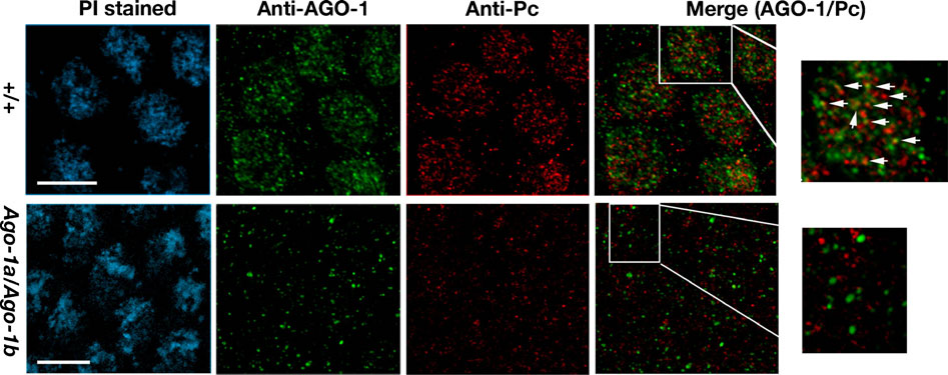
Colocalization of AGO1 and Polycomb proteins in *Drosophila* embryos. Embryos (stage 5) were stained with AGO1 (*green*) and POLYCOMB (Pc) (*red*) antibodies and propidium iodide (DNA, *blue*). Images were taken with 100X objective. Projection of merge image of a single nucleus with higher magnification from each panel showed colocalization of AGO-1 and PC proteins. *Arrows* indicate the overlapping sites (*yellow*) in the nuclei

As noted earlier, non-homologous silencing produced by reciprocal *Adh-w* and *w-Adh* transgenes was repressed by the recruitment of the Pc-G proteins in the *Adh-w* insertion sites (Pal-Bhadra et al. 1999). To reinvestigate, a direct association of Pc bodies and AGO-1 proteins in the embryonic nuclei, we analysed the colocalization of AGO-1 (Kataoka et al. 2001) and Pc-G (Grimaud et al. 2006) proteins by immunostaining (Fig. 8). In wild-type embryos, AGO-1 proteins form uniformly distributed nuclear bodies with 10–15 discrete foci (Fig. S3; stage 5). In contrast, Pc-G proteins, as reported earlier, form a large number of similar nuclear bodies in certain sub-nuclear regions (Fig. 8). In double immunolabelling experiments in the nuclei, 20–25% of the AGO-1 foci were colocalized with Pc-G nuclear bodies, which are apparently 200–500 nm in size. The colocalization between Pc-G and AGO-1 foci suggest that AGO-1 is associated with a subset of Pc-G bodies although Ago-1 is not an integral part of the PRC1 complex.

**Fig. 9:**
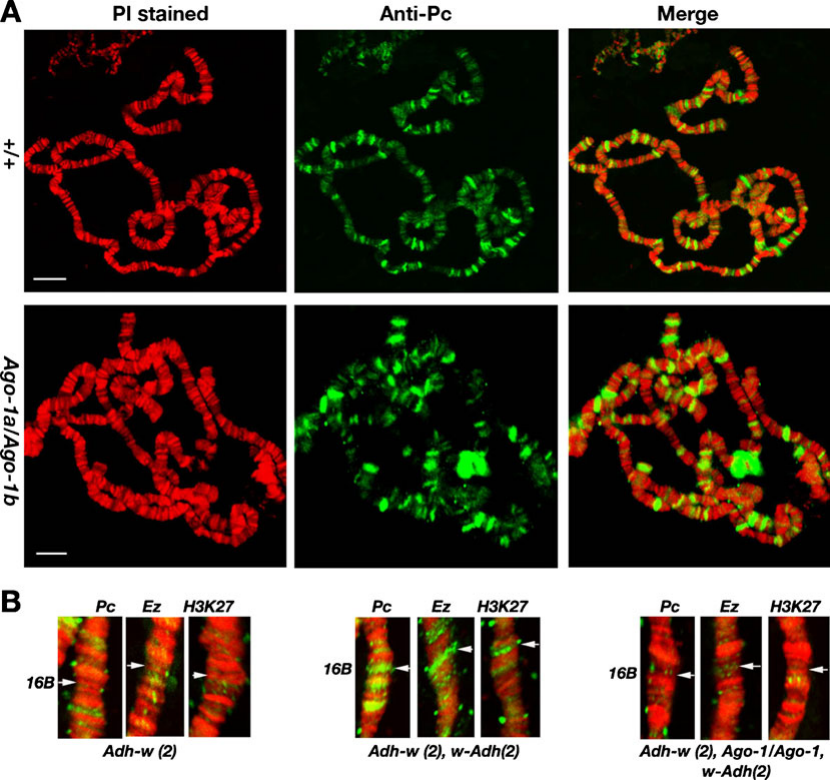
Localization of the PC, Ez and H3K27 (H3me3K27) proteins on the *Adh-w* insertions sites was determined by the immunostaining of the polytene chromosomes. The location of the *Adh-w* gene (16B) was determined by in situ hybridization. **a** The PC binding of the polytene chromosomes in the wild-type and *Ago-1a/Ago-1b* heteroallelic larvae was determined by immunostaining. Scale 10 μm. **b** The enlarged view of the chromosomal segment showing recruitment of the PC, Ez and H3me3K27 methylation in the *Adh-w* sites (16B). The immunostained X chromosomal segment of the 16B region from the *Adh-w/Adh-w* larvae were compared with homozygous *Adh-w* larvae carrying two copies of the reciprocal *w-Adh* transgenes in the wild-type and heteroallelic *Ago-1* (*Ago-1a/Ago-1b*) mutant background. Inclusion of two copies *w-Adh* transgene recruits the proteins at the same site. In contrast, the presence of the heteroalleleic *Ago-1* mutation (*Ago-1a/Ago-1b*) disrupts the association of all three proteins from the *Adh-w* insertion site

Since AGO-1 protein overlaps with Pc-G bodies in embryos, we were interested to examine whether AGO-1 and Pc-G proteins show an overlapping binding on the transgene insertion sites in the larval salivary gland chromosomes. Unfortunately, with repeated attempts, AGO-1 antibody failed to bind on the polytene chromosomes even at a higher concentration. Therefore, only Pc-G bindings at the *Adh-w* insertion site were tested in different genetic backgrounds. We compared Pc, Ez and H3me3K27 bindings on the *Adh-w* insertion site (16B) of larvae carrying two copies of *Adh-w* transgenes showing normal *Adh-w* expression (*Adh-w/Adh-w*) or larvae having two copies of the reciprocal *Adh-w* and *w-Adh* construct exhibiting cosuppressed state (*Adh-w/Adh-w*; *w-Adh/w-Adh*). The *Adh-w* silencing was strongly correlated with the binding of Pc, Ez and H3me3K27 at the 16B site (Fig. 9). We further analysed the antibody staining of the polytene chromosome from the *Ago-1* heteroallelic mutant larvae carrying two copies of each reciprocal transgenes (*Adh-w/Adh-w*; *w-Adh/w-Adh*). A significant disruption of binding of Pc, Ez and H3me3K27 on the *Adh-w* site was noticed indicating repression of transgene cosuppression. However, overall pattern of Pc binding throughout polytene nuclei in wild-type and heteroallelic *Ago-1* larvae were identical. This clearly indicates that wild-type AGO-1 protein is required for the proper recruitment of the epigenetic factors that are prerequisite for the Pc-G binding at the silenced insertion sites.

## Discussion

In the present study, *Drosophila Ago-1* plays an important role in two types of transcriptional silencing. The *Ago-1* mutation impair *w-Adh/Adh/Adh-w* silencing disrupting Pc-G, Ez and H3meK27 proteins that forms a silencing complex to change the histone tail modification in the localized chromatin structure. On the other hand, loss of H3me2K9 methylation by the same mutation suggests a definitive role in H3me2K9 dependent heterochromatin like structure formation in the *mini-w* arrays. A similar result has been reported for the *piwi* mutation (Pal-Bhadra et al. 2002, 2004). In transcriptional silencing, *Ago-1* acts as a factor for guiding the Pc-G silencing complex to transgene sites as well as methylation of the lysine residues in the histone tails during heterochromatin assembly. These results suggest that *Ago-1* interconnects two types of transcriptional silencing; repeat-induced silencing or heterochromatin formation and transgene cosuppression apart from its role in the microRNA processing.

Two members of the *piwi* subfamily, *aubergine* and *piwi* contribute to transgene silencing, chromatin structure and genome stability. They have been shown to be critical for delocalization of histone lysine 9 methylation and heterochromatin formation (Pal-Bhadra et al. 2004). Here, the loss of AGO-1 protein reduces the chromosomal distribution of H3me2K9 and H3me3K27 methylation. These findings strongly suggest that *Ago-1* is an RNAi effector molecule required for chromatin structure, transcriptional repression of the multiple regulatory target sites per se (Grimaud et al. 2006). It probably functions in parallel to *piwi* action specially by recruiting the silencing complex at the *Adh-w* sites (Pal-Bhadra et al. 1999). Therefore, members of the same *Ago-1* family offer a distinct contribution for rendering TGS at various steps (Meyer et al. 2006).

Depletion of PIWI protein abolishes transgene silencing equally by eliminating *Pc-G* binding at PRE and non-PRE target sites (Pal-Bhadra et al. 2002; Grimaud et al. 2006). In contrast, *Ago-1* only shows a consistent disruption of *Pc-G* accumulation from the cosuppressed non-PRE transgenes but not from PRE containing elements (Grimaud et al. 2006). The differential effects of *Ago-1* in PRE and non-PRE transgenes also suggests that *Ago-1* and *piwi* act in a partially redundant manner to control key recruitment of *Pc* proteins in the transgene insertion sites and *piwi* may perform a major function in TGS irrespective of its involvement with a PRE and non-PRE transgenes compared to *Ago-1*.

Moreover, a connection between Pc-G proteins and endogenous siRNA in TGS has recently been established (Zhao et al. 2008). The Polycomb proteins are targeted by a novel species of noncoding RNA generated from the repetitive sequence of the *Xist* locus in mammals (Zhao et al. 2008). In *Drosophila* clustering of PRE targets by the Pc-G proteins on the regulatory *Fab*-7 element of *Abdominal b* gene was assisted by the small species of non-coding RNAs (Grimaud et al. 2006). It is reasonable to argue that endogenous siRNAs are processed by *Ago-1* but may play a partial instructive role in regulating *Polycomb* dependent transcriptional silencing and modulation of chromatin structure. Therefore, *Ago-1* mutations dislodge Pc-G silencing complex by eliminating specific siRNA.

Argonautes are involved in distinct steps of small RNA maturation and small RNA-mediated gene expression by interacting with diverse protein complexes (Ghildiyal and Zamore 2009). Ago-1 in *Arabidopsis* serves as an RNAi processor that recruits tasiRNA, rasiRNA processed from repetitive sequences (Agarwal et al. 2007). Recent studies showed that *Ago-1* and/or *Ago-2* complexes in *Drosophila* were required for dicing of intermediate siRNA and pre-microRNA based on the selection of their structure (Diederichs and Haber 2007). It was our interest to determine whether *Ago-1* might be operating in selecting siRNA and miRNA in the TGS silencing pathways. However, in some cases, *Ago-1* is not the only component for cosuppression and heterochromatin to occur, but some specific endogenous siRNA or miRNA-mediated function are required that directly control the immediate components of chromatin bound silencing complex. It is unlikely that miRNA plays any role in transgene silencing though studies establish that miRNA have some specific function for the transcriptional silencing (Kim et al. 2006). We also, however, note that until further study is performed, it remains a formal possibility that mature miRNA might control Polycomb binding and transcriptional silencing in animals as reported in *Arabidopsis* (Schubert et al. 2005).

Previously, it has been shown that three major components of nuclear RNAi pathway (*piwi*, *spindle E*, *aubergine*) together with heterochromatin-specific proteins are required for the normal organization of the *mini-w* repeats (Pal-Bhadra et al. 2004). *Ago-1* is one of the recent additions in this family. ChIP and immunofluorescence data clearly indicate that H3me2K9 and H3me3K27 levels in the chromatin are reduced in the *Ago-1* mutant flies. These findings demonstrate an important role of *Ago-1* in establishing the pattern of histone tail modifications that directly regulate nuclear organization by affecting chromatin structure and concomitant gene silencing in condensed track of repetitive sequences.

We also observed that *Ago-1* regulates only H3me2K9 and H3me3K27 level significantly. It is reasonable to believe that *Ago-1* and other RNAi components may direct H3K9 methylation through siRNA pathways in *Drosophila* at least in *mini-w* array*.* Conversely, the stability of other repeats was reported to be regulated by combining *Su(var)3-9* and H3K9 methylation that are independent of the RNAi or siRNA pathways (Peng and Karpen 2007). Therefore, overall distribution of post-translation histone modifications in *Drosophila* polytene nuclei were also strikingly different from the *Su(var)3-9* and *Ago-1* mutants. Only H3me2K9 levels were reduced to a considerable level in the *Su(var)3-9* mutant nuclei, although visible amounts were retained in the heterochromatin, fourth chromosome and telomeric region (Schotta et al. 2002). In contrast, reduction in the levels of H3me2K9 and H3me3K27 proteins are less in the *Ago-1* mutants in the diploid cells but mislocalized from the normal chromosomal position. These findings in *Drosophila* are surprising as H3me2K9 is found only at a trace amount in the RNAi mutants in *Schizosaccharomyces pombe* *.*

The functional diversity of the AGO-1 protein in TGS might shed some light on how *Ago-1* functions in small RNA-dependent and RNA-independent transcriptional gene silencing pathways. In conclusion, these studies identify specific functions of AGO-1 to understand their combinatorial role in chromatin modification and heterochromatin formation and its capacity to interact with different species of small regulatory RNAs.

## Electronic supplementary material

Below is the link to the electronic supplementary material.

ESM 1 (DOC 622 kb)

## Abbreviations

Adh: Alcohol dehydrogenase
AGO: Argonaute
CS: Canton S
ChIP: Chromatin-Immunoprecipitation
CYO: Curly
FITC: Fluorescein isothiocyanate
FISH: Flourescent Insitu Hybridisation
EGTA: Ethylene glycol-bis(2-aminoethylether)-N,N,N′,N′-tetraacetic acid
H3K9: Histone H3 lysine 9
HP1: Heterochromatin Protein 1
miRNA: MicroRNA
mRNA: messenger RNA
mw: Miniwhite
NCBI-BLAST: NCBI-Basic Local Alignment Search Tool
OD: Optical density
PBT: PBS +0.1% tween 20
PBS: Phosphate buffered Saline
Pc-G: Polycomb group
PCR: Polymerase Chain Reaction
P-element: P transposon element
piRNA: Piwi interacting RNA
PIWI: P-element induced wimpy testis
PTGS: Posttranscriptional gene silencing
PRE: Polycomb response element
RNAi: RNA interference
rRNA: Ribosomal RNA
RT: Reverse Transcription
SiRNA: Small Interfering RNA
SWI6: SWItch 6
TGS: Transcriptional gene silencing
