# Supplementary material for: Drosophila Argonaute-1 is critical for transcriptional cosuppression and heterochromatin formation
Source: Chromosome Res. 2012 Apr 3;20(3):333–51. doi: 10.1007/s10577-012-9279-y (PMC3323821; doi:10.1007/s10577-012-9279-y)
Supplement: Supplementary file 1 — (DOC 622 kb) [file 10577_2012_9279_MOESM1_ESM.doc]

**Supplementary Online Materials**

***Drosophila Argonaute-1* is critical for transcriptional gene silencing and heterochromatin formation**

**Sreerangam NCVL Pushpavalli*^a^*, Indira Bag*^b^*, Manika Pal-Bhadra*^b^* and Utpal Bhadra*^a*^***

*^a^*Functional genomics and Gene Silencing Group, Centre for Cellular and Molecular Biology, Hyderabad -500007, India.

*^b^*Centre for Chemical Biology, Indian Institute of Chemical Technology, Hyderabad-500007,Indi

Materials and Methods

Genetic crosses: Virgin females carrying *w-Adh* transgene (*y, w; +/+; w-Adh/w-Adh*) on the chromosome 3 were crossed to *yw^67C23^/Y; Gla/CyO; MKRS/TM3Ser* males. Simultaneously, in a separate cross virgin *y w, Ago1b/Cy, +/+* females were also mated to the *yw^67C23^/Y; Gla/CyO; MKRS/TM3Ser* males. The progeny that carried only one balancer marker on each autosome was selected. Further *y w/y w; +/CyO; w-Adh/MKRS* or *y w/y w; In(2LR) Gla/+; w-Adh/ TM3 Ser* females from the former crosses were mated to the males from the later crosses of *y w/Y*; *Ago-1b/Gla; +/TM3Ser* to generate *Ago-1b/CyO; w-Adh/w-Adh* flies.

To combine *Ago-1a* allele with the *Adh-w* transgene, virgin *Basc* females were crossed to the multiple balancer *yw^67C23^/Y; Gla/CyO; MKRS/TM3Ser* males. Flies with balancer markers heterozygous for all three chromosomes were selected. F1 females *Basc/+; Ago1a/In(2LR)Gla; MKRS/+* were generated from a cross between *Basc/Y; In (2LR)Gla/+; MKRS/+* males with *y w*/*Y*; *Ago1a/CyO; +/+* virgin females. Simultaneously, virgin females carrying *Adh-w* on the *X* chromosomes *Adh-w/Adh-w; +/+; +/+* were crossed to *Basc/Y; CyO/+; TM3Ser/+* males obtained from the first cross to generate *Adh-w/Y; CyO/+; TM3 Ser/+.* Virgin *Basc/_+_; Ago-1a/In(2LR)Gla; MKRS/+* females were crossed to the *Adh-w/Y; CyO/+; TM3 Ser/+* males to generate the final *Adh-w/Adh-w; Ago-1a/CyO* stock*.*

Results

Characterization of existing *P* element insertion mutation

As reported earlier, in *l(2)k08121* stock, a *P* element was inserted at 10 nucleotides upstream from the predicted *Argonaute-1* transcripts [*l(2)k08121*] (Fig. 1A, Kataoka et al. 2001). To verify the insertion site of the *P (lacW)* element, a region covering *P* element site of the *Ago-1* promoter was amplified from the genomic DNA of the *l(2)k08121* flies as described in Materials and Methods. The alignment of the amplified sequences with *P (lacW)* and a region of insertion sites demonstrated that the location of the *P (lacW)* is different from the preexisting insertion sites as reported (Katoaka et al. 2001). Our results showed that *P (lacW)* inserts exactly 5 bp (-5) upstream from the *Ago-1* transcription start site instead of 10 bp away from the predicted *Ago-1* transcripts (Fig. 1A).

Generation of *Ago-1* excision lines

We have generated a series of *Ago-1* mutant stocks by excising the *P*-element from the promoter. It was excised by crossing to a stable transposase stock (*w^-^/w^-^ +/+ Δ2-3 Sb/Δ2-3 TM2*). The *P* element has a natural tendency to excise intact element precisely without leaving any fragment of the *P* feet at the insertion site. Here, we found that in many cases, a part of the flanking inverted repeats of the *P*-element end was left behind at the *Ago-1* promoter. Inclusion of such fragments at the site of insertion appears to interfere with *Ago-1* promoter activity. As a result, a loss of function mutation was produced. The genetic scheme for generating a series of *Ago-1* mutations by imprecise excision of a *P* element is summarized in Fig. S1. Females of natural transposase stock (*w^-^/w^-^ +/+ Δ2-3 Sb/Δ2-3 TM2*) were crossed initially to the males carrying balancers for chromosome 2 and 3 (*yw/Y, In(2LR)Gla/CyO, MKRS/TM3 Ser*). F1 *w^-^/y w; CyO/+; Δ2-3 Sb/TM3 Ser* males were selected and further back crossed to the virgin females of a parental *P* stock (*y w/y w; Ago1a/CyO; +/+*) to generate excision flies in the presence of active transposase. The progeny males with mosaic red eye color (*w^-^/Y, Ago-1a/CyO; Δ2-3 Sb/+*) were selected and further test crossed with females carrying two complementary second chromosomal balancers (*w^-^/w-; Pin/CyO; +/+*). The white-eyed F3 males that are devoid of transposase enzyme source were selected (Fig. S1 and S4) and each male was pair-mated with *y, w* females carrying all autosomal balancer chromosomes (*yw/yw; In(2LR)Gla/SM6a,CyO; MKRS/TM3,Ser*) for generating stable stocks. Initially, we have generated 291 independent excision lines using same crossing schemes.

Molecular characterization of excision lines

Genomic DNA from all sixty-seven lines was extracted and PCR amplification was carried out using gene specific and *P* element specific primers as noted above. Two feet of the *P* element along with *Ago-1* flanking sequences were amplified to determine whether the *P* element has left behind any sequences during excision. The results of the amplification at the *P/Ago-1* junction were summarized in Table S1. In few lines (18/67, 28%), sequences did not amplify suggesting that excised lines do not carry complete amplified regions. Therefore a precise *P* excision or unlikely a larger deletion at the *P/Ago-1* junction was expected (Fig. S5-6). In some cases, amplification took place at the both ends of the *P* element, but in forty-four out of the sixty-seven (66.66%) excision lines, amplification occurred at either end of the *P* element. It indicates that at the time of excision, at least one foot of the *P* element was left behind at the *Ago-1*promoter. An alignment of amplified PCR sequences with genomic regions determines the length of the left over elements. On the other hand, in few cases, the existence of two *P* element feet but loss of the *mini-w* phenotype indicated that only the intercalated *mini-w* gene was excised leaving both feet of the *P* element at the insertion site (Fig. S5-6).

**Table S1**. Molecular characterization of sixty seven excision lines.

| S.No | AGO Excision lines | P element left foot | P element right foot |
| --- | --- | --- | --- |
| 1. | CS | - | - |
| 2. | AGO72/CyO | **+** | + |
| 3. | Ex-41 | + | - |
| 4. | Ex-252 | - | + |
| 5. | Ex-27 | + | + |
| 6. | Ex-38 | + | + |
| 7. | Ex-180 | - | + |
| 8. | Ex-144 | - | - |
| 9. | Ex-43 | + | - |
| 10. | Ex-261 | - | - |
| 11. | Ex-162 | + |  |
| 12. | Ex-122 | + | + |
| 13. | Ex-258 | + | + |
| 14. | Ex-52 | + | + |
| 15. | Ex-289 | - | + |
| 16. | Ex-120 | - | - |
| 17. | Ex-96 | + | + |
| 18. | Ex-105 | + | - |
| 19. | Ex-74 | + | + |
| 20. | Ex-42 | + | + |
| 21. | Ex-220 | + | + |
| 22. | Ex-239 | - | + |
| 23. | Ex-55 | + |  |
| 24. | Ex-50 | - | - |
| 25. | Ex-224 | - | - |
| 26. | Ex-174 | - | - |
| 27. | Ex-200 | - | - |
| 28. | Ex-240 | - | + |
| 29. | Ex-142 | + | + |
| 30. | Ex-288 | - | - |
| 31. | Ex-160 | + | + |
| 32. | Ex-143 | + | + |
| 33. | Ex-106 | + | + |
| 34. | Ex-207 | + | + |
| 35. | Ex-26 | + | + |
| 36. | Ex-13 | + | + |
| 37. | Ex-184 | - | + |
| 38. | Ex-66 | - | + |
| 39. | Ex-17 | + | + |
| 40. | Ex-165 | - | + |
| 41. | Ex-57 | - | + |
| 42. | Ex-241 | + | - |
| 43. | Ex-242 | - | + |
| 44. | Ex-45 | + |  |
| 45. | Ex-37 | + | - |
| 46. | Ex-293 | - | - |
| 47. | Ex-33 | - | - |
| 48. | Ex-18 | - | - |
| 49. | Ex-105 | + | + |
| 50. | Ex-196 | - | + |
| 51. | Ex-166 | + |  |
| 52. | Ex-165 | - | - |
| 53. | Ex-28 | + | + |
| 54. | Ex-17 | - | - |
| 55. | Ex-251 | + | + |
| 56. | Ex-209 | + | - |
| 57. | Ex-280 | + | + |
| 58. | Ex-35 | - | - |
| 59. | Ex-250 | + | + |
| 60. | Ex-181 | + | + |
| 61. | Ex-236 | - | - |
| 62. | Ex-228 | + | - |
| 63. | Ex-135 | + | + |
| 64. | Ex-265 | - | - |
| 65. | Ex-232 | - | + |
| 66. | Ex-226 | - | - |
| 67. | Ex-73 | - | + |
| 68. | Ex-231 | - | - |
| 69. | Ex-189 | - | + |

The results of the PCR amplification from the left and right foot of the *P* element from each excision line are summarized. The + and – values represent positive and negative results of the PCR amplification. The PCR fragment was amplified using a *P* element (forward) and a (reverse) gene specific primer from the genomic DNA extracted from each excision line.

**Table –S2** Result of lethality of each *Ago-1* excision line with *Ago-1* deficiency stock [*Df (2R)50C-107/CyO*]*.*

___________________________________________________________________________

Selected excision lines *Ago1a Ex/CyO* *Df (2R)50C-107/Ago1a ^ex^*

or *Df (2R)50C-107/CyO*

1. Ex-28 189 ---

2. Ex-37 146 ---

3. Ex-105 209 ---

4. Ex-189 198 ---

5. Ex-196 221 ---

6. Ex-242 239 ---

___________________________________________________________________________

The number of balancer and non-balancer progeny was counted from three independent crosses for each genotype. Twenty virgin females of each excision stocks were mated to the fifteen heterozygous males of *Ago-1* deficiency stocks.

**Table S3.** Lethality of various heteroallelic combinations of six *Ago-1* excision alleles

-------------------------------------------------------------------------------------------------------------------------------

Ex-28 Ex-37 Ex-105 Ex-189 Ex-196 Ex-242

1. Ex-28 -

2. Ex-37 + -

3. Ex-105 + - -

4. Ex-189 - + - -

5. Ex-196 - + - + -

6. Ex-242 - + - - - -

-------------------------------------------------------------------------------------------------------------------------------

Flies carrying each of the alleles of *Ago-1^ex^* over a marked balancer were crossed to all other *Ago-1^ex^* alleles over a balancer. The progeny flies were separated based on the genotype and scored. Progeny were examined for the absence of balancers indicating heteroallelic escapers. – no escapers, + heterallelic escapers recovered. Results are based on examination of 150-200 progeny from each cross.

**Table S4**. Description and the sequences of RT-PCR and Reverse RT PCR primer sets

Gene Primer Sequences Length product size Primer

_____________________________________________________________________________________

*white* promo 5’ **CCACAGAAATATCGCCGTCT 3’** 20bp 178 bp Forward

*white* promo 5’**GAGAGGAGTTTTGGCACAGC 3’** 20bp 178bp Reverse

*white* 2^nd^exon 5’**TCGCAGAGCTGCATTAACC 3’** 19bp 156bp Forward

*white* 2^nd^exon 5’**ATTGACCGCCCCAAAGAT 3’** 18bp 156bp Reverse

____________________________________________________________________________________

The *mini-w* primers are noted based on the transcriptional start site of the *white* gene. Promo means promoter.


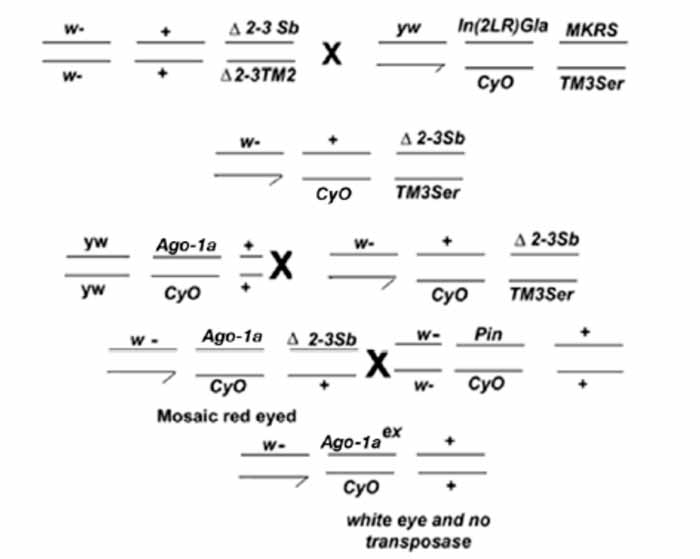


**Fig. S1.** The genetic crosses for generating of *Ago-1* excision stocks by using imprecise excision of a *P* element located at the promoter of the *Ago-1* insertion mutation [*l(2)k08121*] were shown.


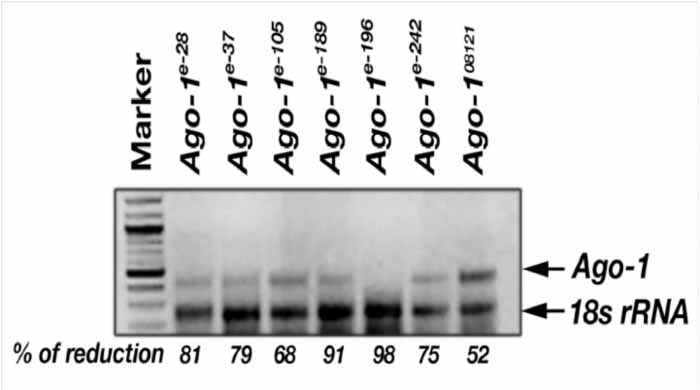


**Fig. S2.**Photographs of an ethidium bromide stained gel showing the relative amount of *Ago-1* mRNA of six selected excision and a parental *P* stock. Quantitative RT-PCR was carried out using *Ago-1* and *18s rRNA* primer sets. The amount of the *Ago-1* and *18s rRNA* transcripts are marked. The name of each line and percentage of reduction relative to the wild type *Ago-1* mRNA are noted at the top and bottom of the gel.


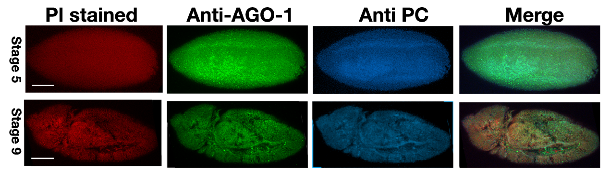


**Fig. S3.** Distribution of AGO-1 and Pc proteins in the wild type *Drosophila* embryos. Only AGO-1 protein was accumulated at a reasonable level during blastoderm stage (stage 5), but both AGO-1 and Pc proteins were localized during the germ band elongation (stage 9).

**
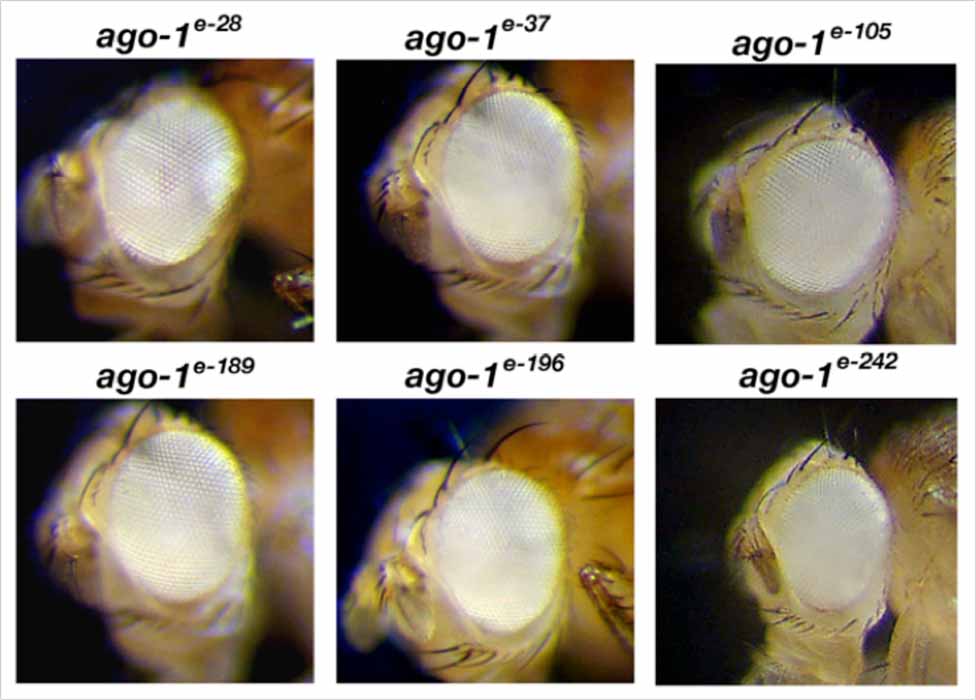
**

**Fig. S4.**  Adult male flies showing *white* minus phenotype after excising a P (*lacW*) element inserted in the promoter of the *Ago-1* gene. The *mini-w* transgene, an integral part of the *P* element construct was deleted from each fly during excision. The name of the excision stocks is noted.

**
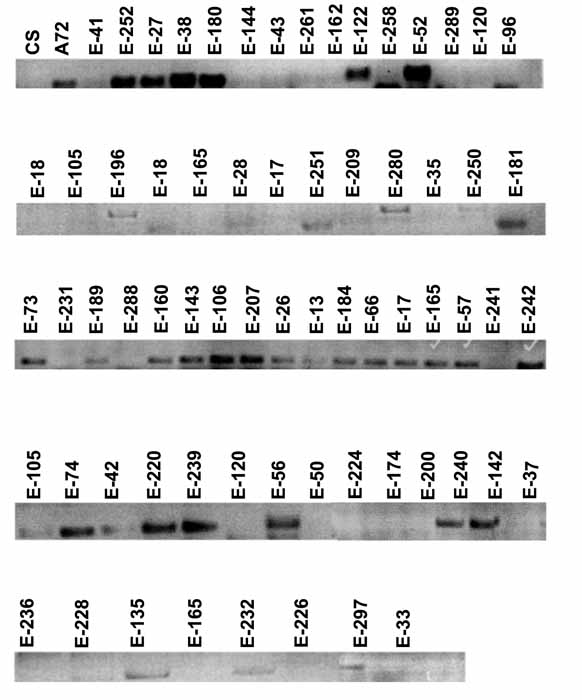
**

**Fig. S5**. Characterization of *Ago-1* excision lines based on PCR amplification. Images of the agarose gel showing fragments amplified by the PCR of DNA extracted from each excision line using forward primer of the *Ago-1* gene and reverse primer of the *P(lacW).*


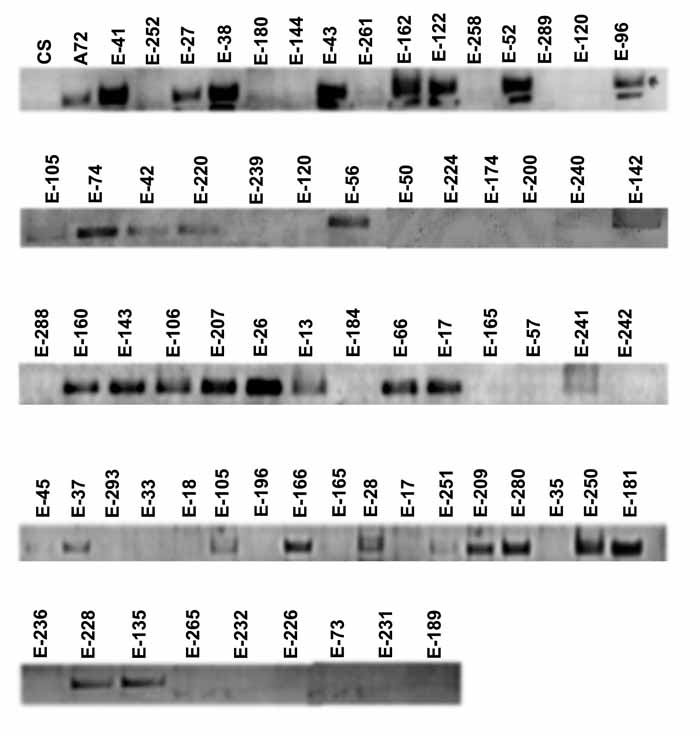


**Fig. S6.** Characterization of *Ago-1* excision lines based on PCR amplification. Images of the agarose gel showing fragments amplified by the PCR of DNA extracted from each excision line using reverse primer of the *Ago-1* gene and forward primer of the *P(lacW).*

**References**

Fanti L, Dorer DR, Berloco M, Henikoff S, Pimpinelli S (1998) Heterochromatin protein 1 binds transgene arrays. Chromosoma 107:286-292

**Huang AM, Rehm Jay E, Rubin GM (2000)** Quick Preparation of Genomic DNA from *Drosophila* Adapted from *Drosophila Protocols* (eds. Sullivan et al.). CSHL Press, Cold Spring Harbor, NY, USA

Kataoka Y, Takeichi M, Uemura T (2001) Developmental roles and molecular characterization of a *Drosophila* homologue of *Arabidopsis* Argonaute1, the founder of a novel gene superfamily. Genes Cells 6:313-325

Kim DH, Saetrom P, Snove Jr O, Rossi JJ (2008) MicroRNA-directed transcriptional gene silencing in mammalian cells. PNAS 105:16230-16235

Pal-Bhadra M, Bhadra U, Jackson DE, Mamatha L, Park P, Shukla SD (2007) Distinct methylation patterns in histone H3 at Lys-4 and Lys-9 correlate with up- & down-regulation of genes by ethanol in hepatocytes. Life Sci 81:979–987
